# The role of PlsC in *Brucella melitensis* virulence: impacts on membrane homeostasis, stress tolerance, and pathogenesis

**DOI:** 10.1186/s13567-026-01811-0

**Published:** 2026-07-03

**Authors:** Fazhi Xu, Yao Feng, Mengsi Li, Na Li, Guangyu Yang, Jing Qu, Zheliang Yang, Yang Li, Shaohui Wang, Yanqing Bao, Jingjing Qi, Mingxing Tian

**Affiliations:** 1 College of Veterinary Medicine, Anhui Agricultural University, Hefei, 230036 China; 2 Shanghai Veterinary Research Institute, Chinese Academy of Agricultural Sciences (CAAS), 518 Ziyue Road, Shanghai, 200241 China

**Keywords:** *Brucella melitensis*, *plsC*, phosphatidic acid, membrane homeostasis, virulence

## Abstract

*Brucella melitensis*, a facultative intracellular pathogen, relies on membrane integrity and homeostasis to resist host defenses and establish infection. The *plsC* gene encodes 1-acyl-sn-glycerol-3-phosphate acyltransferase, a key enzyme in the glycerophospholipid pathway that catalyzes the synthesis of phosphatidic acid, an essential precursor for membrane lipid formation. However, its role in *B. melitensis* virulence remains poorly understood. Here, we constructed a *plsC* deletion mutant (Δ*plsC*) and a complemented strain (Δ*plsC*-Com) in *B. melitensis* strain M5 and characterized their phenotypes. Deletion of *plsC* impaired bacterial growth in nutrient-limited media, reduced tolerance to hydrogen peroxide and polymyxin B, and decreased lipid synthesis while increasing outer membrane permeability. Ultrastructural analysis revealed surface roughness, cytoplasmic voids, and nucleoid condensation in the mutant. Although Δ*plsC* retained normal adhesion and invasion capabilities in RAW264.7 macrophages and HeLa cells, its intracellular survival was specifically attenuated in macrophages at 48 h post-infection. In a mouse model, Δ*plsC* showed significantly reduced colonization of the spleen and liver and induced fewer and smaller liver granulomas as compared with the parental and complemented strains. These results demonstrate that PlsC is essential for maintaining membrane homeostasis and stress resistance in *Brucella*, which in turn supports its survival within professional phagocytes and full virulence in vivo. Our study suggests a critical link between phospholipid metabolism and *Brucella* pathogenicity.

## Introduction

*Brucella*, a Gram-negative, facultative intracellular pathogen, poses a significant economic concern in agriculture and veterinary fields, owing to its ability to cause abortion and infertility in livestock including goats, sheep, cattle, pigs, and dogs [1]. Moreover, transmission to humans occurs through contact with infected animals or contaminated animal products, causing a chronic debilitating febrile illness known as brucellosis. The infection often follows a persistent course and may even last a lifetime in some cases [2]. Recognized as one of the most widespread zoonoses globally [3], the long-term persistence of *Brucella* is largely attributed to its capacity to survive and replicate within host macrophages [4].

Belonging to the class Alphaproteobacteria, *Brucella* species are nonspore-forming coccobacilli that are traditionally considered nonmotile; however, several published studies have revealed that they possess a cryptic flagellum which can be expressed under specific environmental conditions [5]. Their pathogenicity relies on the ability to adapt to harsh intracellular microenvironments—such as low oxygen, acidic pH, and nutrient scarcity—to enable survival and proliferation inside host cells [6]. Notably, *Brucella* lacks classic virulence factors including exotoxins, cytolysins, and capsules [7]. Instead, it employs a combination of regulatory systems and virulence determinants to evade host immune clearance and establish intracellular niches [4, 6]. Key virulence factors include lipopolysaccharide (LPS), the type IV secretion system (T4SS), and the two-component regulatory system BvrR/S [7]. Furthermore, bacterial membrane composition is a critical determinant of virulence, contributing to resistance against antimicrobial peptides and the suppression of phagocytosis [8]. Phospholipids, the main lipid constituents of the cell membrane, are synthesized via the glycerophospholipid pathway. Phosphatidic acid (PA) serves as an essential precursor for all major phospholipid species—such as phosphatidylethanolamine, cardiolipin, and phosphatidylglycerol—and directly regulates the homeostasis of membrane lipid composition [9].

The *plsC* gene (encoding 1-acyl-sn-glycerol-3-phosphate acyltransferase) plays a central role in bacterial phospholipid biosynthesis by catalyzing the second acylation step in PA synthesis, converting lysophosphatidic acid to PA, thereby determining the fatty acid composition of membrane phospholipids and influencing membrane physical properties [10]. In *Brucella*, Barbieux et al. identified, via transposon sequencing, that a *plsC* deletion in *B. melitensis* impaired bacterial colonization of the spleen and lungs of mice [11]; however, the underlying mechanisms remain unclear.

To investigate the role of *plsC* in *Brucella* pathogenesis, we constructed a *plsC* deletion mutant and a complemented strain in *B. melitensis*. Using a series of experiments, we demonstrated that the absence of *plsC* attenuates bacterial growth under nutrient-limited conditions, reduces tolerance to hydrogen peroxide and polymyxin B, impairs lipid synthesis, disrupts membrane homeostasis, and diminishes virulence in mice. This study provides an analysis of how *plsC* deletion affects *Brucella* virulence and offers new insights into the relationship between lipid metabolism and pathogenicity in *Brucella*.

## Materials and methods

### Strains, cells, and culture conditions

The attenuated *B. melitensis* vaccine strain M5 (China Veterinary Culture Collection Center, Beijing, China) and its derivatives were cultured in tryptic soy broth (TSB; BD, Franklin Lakes, NJ, USA) or on tryptic soy agar (TSA) at 37 °C under 5% CO_2_. All experiments involving live low virulence *B. melitensis* strains were conducted in a Biosafety Level 2 (BSL-2) facility at the Shanghai Veterinary Research Institute (SHVRI), Chinese Academy of Agricultural Sciences (CAAS). *Escherichia coli* DH5α (TIANGEN, Beijing, China) was grown in Luria–Bertani (LB) medium at 37 °C. The murine monocyte-macrophage cell line RAW264.7 (TIB-71, ATCC) and HeLa cells (CCL-2, ATCC) were maintained in Dulbecco’s modified eagle medium (DMEM; Gibco, Waltham, MA, USA) supplemented with 10% fetal bovine serum (FBS; TransGen BioTech, Beijing, China) at 37 °C in a 5% CO_2_ atmosphere. All strains and plasmids used in this study are listed in Table 1.

**Table 1:** **Bacterial strains and plasmids used in this study**

Strains and plasmids	Description	Sources
Strains		
*B. melitensis* M5	Low virulence strain; smooth phenotype	CVCC
Δ*plsC*	The *plsC* deletion mutant derived from M5	This study
Δ*plsC*-Com	The *plsC* complemented strain	This study
*E. coli* DH5α	F^−^, φ80d*lacZ* ΔM15, Δ*(lacZYA-argF)*U169, *recA1*, *endA1*, *hsdR17(rk*^*−*^*, mk*^+^*)*, *phoA*, *supE44*, *thi-1*, *gyrA96*, *relA1*, *λ*^*−*^	TIANGEN
Plasmids		
pKB	Kan^R^; pUC19 derived plasmid containing *sacB* gene	[12]
pMiniTn7TK	Kan^R^; miniTn7 vector	[12]
pHelp1	Amp^R^; Tn7 transposase TnsABCD	[12]
pKB-Δ*plsC*	pKB plasmid carrying the upstream and downstream homologous arm fragments of the *plsC* gene	This study
pMiniTn7TK-C*plsC*	pMiniTn7TK plasmid carrying the *plsC* gene	This study

### Plasmid construction

The suicide plasmid (a nonreplicative vector that cannot propagate in *Brucella*, thus enabling homologous recombination-mediated gene deletion) was constructed as previously described [12]. Briefly, the upstream and downstream homologous arms of the *plsC* gene were amplified from the genomic DNA of *B. melitensis* M5 using primer pairs PlsC-UF/UR and PlsC-DF/DR, respectively. Polymerase chain reaction (PCR) products were separated by agarose gel electrophoresis and purified using a gel extraction kit (TIANGEN). The purified fragments were fused by overlap PCR with primers PlsC-UF/DR, and the resulting fusion fragment was purified. The fusion fragment was then ligated into the linearized pKB vector using the ClonExpress II One Step Cloning Kit (Vazyme, Nanjing, China) and transformed into *E. coli* DH5α, yielding the suicide plasmid pKB-Δ*plsC*.

The complementation plasmid was constructed on the basis of a reported method [12]. The complementation fragment was amplified from *B. melitensis* M5 genomic DNA using primers Com-F and Com-R. Using the same cloning strategy as above, the fragment was ligated into the linearized pMiniTn7TK plasmid and transformed into *E. coli* DH5α, resulting in the complementation plasmid pMiniTn7TK-C*plsC*. All primers used in this study are listed in Table 2.

**Table 2:** **Primers used in this study**

Primer name	Sequences (5′–3′)
PlsC-UF	GGTACCCGGGGATCCTTGCAGCCGCACAAGCGGAG
PlsC-UR	AGTTCGACATCGCCCTCATTGCGCCCTCTTCAGGT
PlsC-DF	AAGAGGGCGCAATGAGGGCGATGTCGAACTGATGC
PlsC-DR	TGCCTGCAGGTCGACCCGGCAGATGCTCAAACTTG
Com-F	CATGAGCTCACTAGTGGATCCCTGATTGCGCAGGATACCAT
Com-R	GCAAGGCCTTCGCGAGGTACCCAGCACCAGATCGGCATCGT
In-PlsC -F	TCGCATAAGAACGGTCGGTA
In-PlsC -R	GAAAAGCGCAGTCTTGAACG
Out-PlsC -F	GCCCCGCCTATTATGAAACG
Out-PlsC -R	GAAGGTGACAATCTGCTCGG

### Construction of deletion and complemented strains

The deletion mutant and complemented strain were generated as previously described [12]. Briefly, *B. melitensis* M5 was cultured to early-log phase (OD_600_ = 0.4–0.8), chilled on ice for 10 min, washed twice with sterile deionized water, and resuspended in 1/10 volume of 10% glycerol to prepare electrocompetent cells. Then, 2 μg of the suicide plasmid pKB-Δ*plsC* was electroporated into the competent cells at 2.4 kV, 400 Ω. After electroporation, cells were recovered in 1 mL of prewarmed TSB for 2 h at 37 °C with shaking and then plated on TSA containing 50 μg/mL kanamycin for primary selection. Single colonies were cultured in TSB and subsequently plated on TSA supplemented with 5% sucrose for secondary screening. Deletion of the *plsC* gene was confirmed by PCR, and the resulting mutant was designated Δ*plsC*.

For complementation, 1 μg of pMiniTn7TK-C*plsC* and 1 μg of the helper plasmid pHelp1 were co-electroporated into Δ*plsC* competent cells. Transformants were selected on TSA plates containing kanamycin and verified by PCR. The complemented strain was named Δ*plsC*-Com.

### Growth curve analysis

The growth kinetics of *Brucella* strains were assessed in both nutrient-rich and chemically defined media. Overnight cultures were harvested at mid-log phase, washed twice with phosphate-buffered saline (PBS: 137 mM NaCl, 2.7 mM KCl, 10 mM Na_2_HPO_4_, 2.0 mM KH_2_PO_4_, pH 7.4, 1 × working solution; Servicebio, Wuhan, China), and adjusted to an OD_600_ of 1.0. Bacterial suspensions were then diluted 1:10 into fresh TSB or into Plommet’s medium (PM) [13]. The PM medium contained the following basal components per liter: 2.3 g K_2_HPO_4_, 3 g KH_2_PO_4_, 0.1 g Na_2_S_2_O_3_, 5 g NaCl, 0.2 g niacin, 0.2 g thiamine, 0.07 g pantothenate, 0.5 g (NH_4_)_2_SO_4_, 0.01 g MgSO_4_, 0.1 mg MnSO_4_, 0.1 mg FeSO_4_, 0.1 mg biotin, and 1 mM methionine. To evaluate carbon source utilization, the medium was supplemented individually with one of the following carbon sources at the indicated final concentration: 2 g/L *meso*-erythritol, 1 g/L D-glucose, or 2 g/L L-fucose. All cultures were incubated at 37 °C with shaking at 220 rpm. Bacterial growth was monitored by measuring the OD_600_ at 6-h intervals throughout the experiment. Each condition was tested in triplicate, and growth curves were plotted on the basis of the mean OD_600_ values over time.

### Stress tolerance assays

Stress tolerance assays were performed as previously established [12]. To evaluate bacterial survival under various stress conditions, hydrogen peroxide (H_2_O_2_), polymyxin B, sodium dodecyl sulfate (SDS), and acid tolerance assays were conducted. Briefly, the M5, Δ*plsC*, and Δ*plsC*-Com strains were grown to OD_600_  ≈1.0 and diluted to appropriate concentrations in sterile PBS (1 ×, pH 7.4).

For H_2_O_2_ and polymyxin B tolerance, bacterial suspensions (5 × 10^5^ CFU/mL) were mixed with an equal volume of stressor (final concentrations: 2 mM H_2_O_2_ or 2 mg/mL polymyxin B) and incubated at 37 °C for 1 h. After serial dilution and plating, survival rates were calculated as (CFU of treated group/CFU of the PBS control group) × 100%.

SDS tolerance was assessed using a disk diffusion assay. Bacterial lawns were prepared by spreading 100 μL of the suspension (5 × 10^9^ CFU/mL) onto TSA plates. A sterile disk containing 7 μL of 20% SDS was placed in the center, and the diameter of the inhibition zone was measured after incubation to evaluate membrane integrity.

Acid tolerance was tested by exposing bacteria to 0.1% peptone solution adjusted to pH 4.5 for 2 h. Bacteria treated with pH 7.2 buffer served as a control. Survival rates were determined by plating and colony counting. All experiments were performed with three biological replicates.

### Fluorescent dye uptake assays

To assess membrane integrity and permeability, fluorescent dye uptake assays were conducted using Nile red (NR), propidium iodide (PI), and N-phenyl-1-naphthylamine (NPN) [12]. Briefly, *B. melitensis* strains M5, Δ*plsC*, and Δ*plsC*-Com were cultured to mid-log phase, washed twice with PBS (1 ×, pH 7.4), and adjusted to OD_600_ = 1.0. Then, 200 µL of each bacterial suspension was dispensed into wells of a 96-well black plate (Corning, Corning, NY, USA). For NR uptake, 2.5 µL of 5 mM NR was added per well, followed by incubation in the dark for 1 h, after which fluorescence was measured at excitation/emission wavelengths of 552/636 nm. For PI uptake, 2 µL of 1 mM PI was added per well, incubated for 1 h in the dark, and fluorescence was recorded at excitation/emission wavelengths of 535/617 nm. For NPN uptake, 2 µL of 1 mM NPN was added per well and fluorescence was measured after 10 min of incubation at excitation/emission wavelengths of 355/460 nm. Each strain was assayed in triplicate with 3–5 technical repeats per experiment, and all assays were independently performed three times.

### Scanning electron microscopy (SEM)

Bacterial cells at mid-logarithmic growth phase were harvested by centrifugation, washed twice with PBS (1 ×, pH 7.4), and primarily fixed with 2.5% glutaraldehyde. After three washes with PBS, the samples were postfixed with 1% osmium tetroxide (in PBS) for 1–2 h at room temperature in the dark. Following another three washes with PBS, the samples were dehydrated through a graded ethanol series (30%, 50%, 70%, 80%, 90%, 95%, and 100% twice), followed by treatment with isoamyl acetate for 15 min. The specimens were then mounted on glass coverslips, dried using a critical point dryer (K850, Quorum Technologies, Lewes, UK), and sputter-coated with gold for approximately 30 s using an ion sputter coater (MC1000, Hitachi High-Tech, Tokyo, Japan). Finally, the samples were imaged using a scanning electron microscope (SU8100, Hitachi High-Tech).

### Transmission electron microscopy (TEM)

For TEM sample preparation, bacterial cells collected at mid-logarithmic phase were fixed with 2.5% glutaraldehyde, washed three times with PBS (1 ×, pH 7.4), and then embedded in prewarmed, molten 1% agarose. The agarose-embedded samples were postfixed with 1% osmium tetroxide (in PBS) for 2 h at room temperature in the dark and washed three times with PBS. Dehydration was performed using a graded ethanol series (30%, 50%, 70%, 80%, 95%, and 100% twice), with each step lasting 20 min, followed by two 15-min treatments with 100% acetone. Subsequently, the samples were infiltrated and embedded: they were first infiltrated with a mixture of acetone and 812 epoxy resin (1:1 ratio at 37 °C for 2–4 h, then 1:2 ratio at 37 °C overnight), followed by pure 812 resin at 37 °C for 5–8 h. The samples were then placed in embedding molds and polymerized (37 °C overnight, followed by 60 °C for 48 h) to obtain hardened resin blocks. Ultrathin sections (60–80 nm) were cut from the blocks using an ultramicrotome (UC7, Leica Microsystems, Wetzlar, Germany) and collected on 150-mesh copper grids. The sections were stained with 2% uranyl acetate in saturated ethanol for 8 min in the dark, rinsed with 70% ethanol and ultrapure water, and then counterstained with 2.6% lead citrate (protected from CO_2_) for 8 min. After further rinsing and drying, the sections were observed and imaged using a transmission electron microscope (HT7800, Hitachi High-Tech).

### Cell infection assays

Cell infection assays were adapted from a previously reported method [14]. RAW264.7 or HeLa cells were seeded in 24-well plates and grown to confluence. Cells were infected with *Brucella* at a multiplicity of infection (MOI) of 100 (RAW264.7) or 500 (HeLa) for 1 h at 37 °C (defined as 0 h). After infection, cells were washed three times with PBS (1 ×, pH 7.4) to remove nonadherent bacteria. To quantify adherent bacteria, cells were lysed with 0.25% Triton X-100 in PBS, and lysates were plated on TSA for CFU counting. To assess bacterial invasion, infected cells were treated with DMEM containing 100 μg/mL gentamicin for 1 h at 37 °C to kill extracellular bacteria. After washing, cells were lysed and plated as above to determine intracellular bacterial counts. For intracellular survival analysis, cells were maintained in DMEM supplemented with 1% FBS and 50 μg/mL gentamicin. At 24 and 48 h post-infection, cells were lysed and plated to quantify viable intracellular bacteria. Adhesion, invasion, and intracellular survival were compared among the parental, Δ*plsC*, and Δ*plsC*-Com strains.

### Mouse infection experiment

*B. melitensis* M5, Δ*plsC*, and Δ*plsC*-Com strains were grown in TSB with shaking to mid-log phase, adjusted to OD_600_ = 1.0 (approximately 5 × 10^9^ CFU/mL), and diluted in PBS (1 ×, pH 7.4) to 1 × 10^7^ CFU/mL. Then, 6- to 8-week-old female BALB/c mice were randomly divided into four groups (*n* = 6 per group). Mice were intraperitoneally injected with 0.1 mL of bacterial suspension containing 1 × 10^6^ CFU of the respective strain. The control group received an equal volume of PBS. A total of 2 weeks post-infection, mice were euthanized by cervical dislocation. Spleen and liver tissues were aseptically collected and weighed. Each tissue was homogenized in 3 mL of PBS containing 0.25% Triton X-100. Homogenates were serially diluted, plated on TSA plates, and incubated at 37 °C for 3–4 days. Bacterial loads were expressed as CFU per gram of tissue. Colonization capacities of the different strains in the spleen and liver were compared to evaluate the impact of *plsC* deletion on *Brucella* virulence in vivo. Mouse infections were performed in an Animal Biosafety Level 2 (ABSL-2) laboratory at the SHVRI, CAAS.

### Histopathological analysis

Liver tissues from infected mice were fixed in 4% paraformaldehyde, embedded in paraffin, and sectioned. Hematoxylin and eosin (H&E) staining was performed by Borove Biotechnology Co., Ltd. Whole-slide images were acquired using a Pannoramic MIDI II system (3DHistech, Budapest, Hungary) and analyzed with CaseViewer v2.4 software (3DHistech). For each group, nine regions (10 mm^2^ each) were randomly selected from three liver sections (each from a different mouse) to quantify granuloma density per unit area. Additionally, the diameters of 150 randomly selected granulomas per group were measured, followed by statistical analysis.

### Statistical analysis

Statistical analyses were performed using GraphPad Prism 9.5 (GraphPad Software, San Diego, CA, USA). Comparisons between two groups were conducted using Student’s *t*-test. Multiple-group comparisons were analyzed by one-way or two-way analysis of variance (ANOVA) followed by Dunnett’s post hoc test. A *p*-value < 0.05 was considered statistically significant.

## Results

### Construction of *plsC* deletion and complemented strains in *B. melitensis*

**Figure 1:**
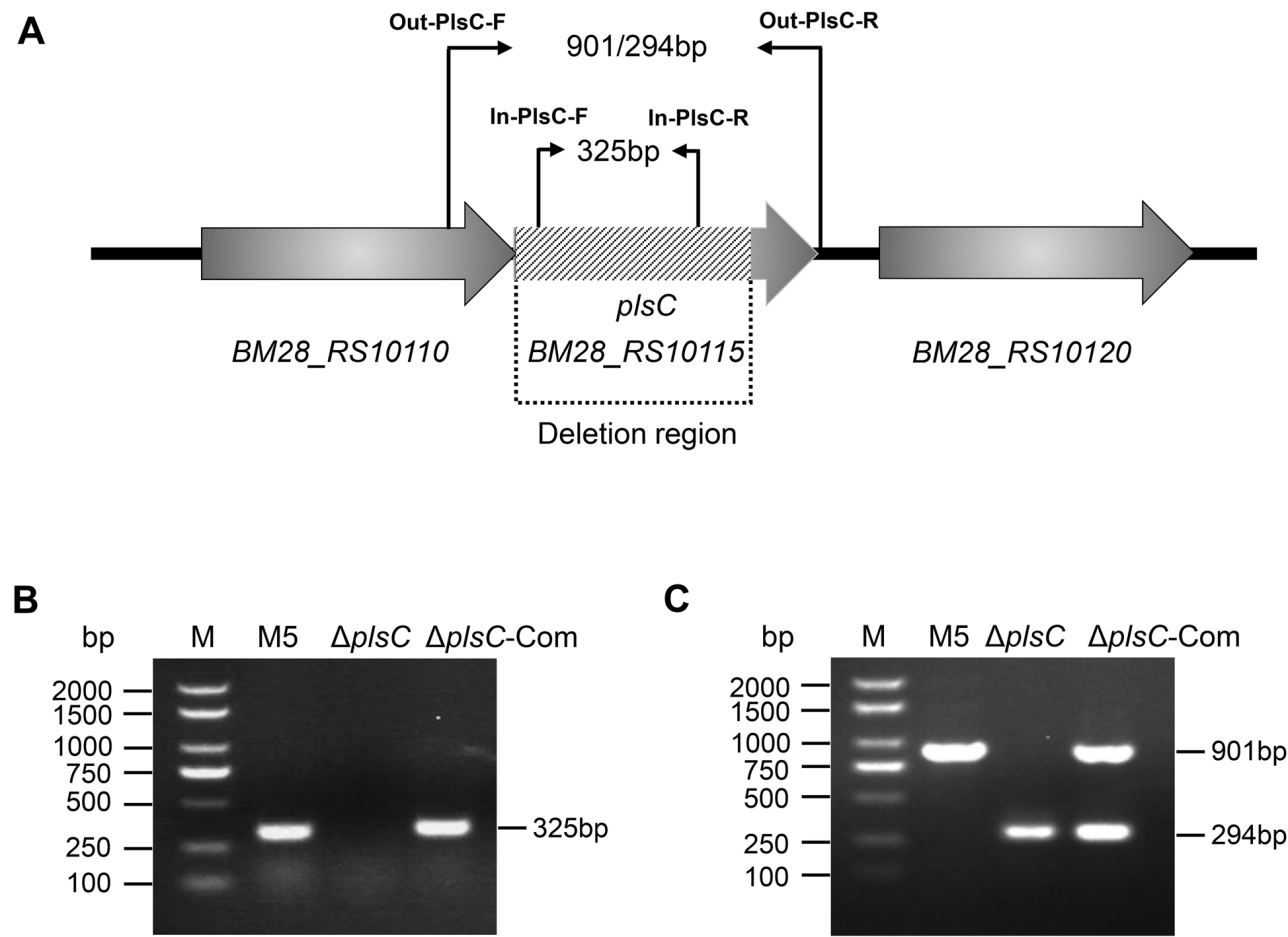
**Construction and verification of the *plsC* deletion and complemented strains.** **A** schematic representation of the *plsC* gene locus and the binding sites of the primers used. **B** PCR verification of the *plsC* deletion mutant and complemented strain using the internal primer pair In-PlsC-F/R. **C** PCR verification using the external primer pair Out-PlsC-F/R.

To investigate the function of the *plsC* gene, we successfully constructed a *plsC* deletion mutant and its complemented strain in *B. melitensis*. The gene is 804 bp long, encoding a protein of 267 amino acids. For the deletion mutant, a 607-bp fragment within the *plsC* open reading frame (ORF), representing 75.5% of its full length, was removed (Figure 1A). The complemented strain was constructed using the pMiniTn7TK plasmid system [12]; a DNA fragment containing the *plsC* ORF along with its predicted promoter and terminator regions was directionally inserted into a specific site between the *glmS* and *recG* genes on chromosome II of the bacterium. The constructed strains were verified by PCR. Using the internal primer pair In-PlsC-F/In-PlsC-R, specific bands of approximately 325 bp were amplified from both the parental and complemented strains, whereas no band was detected for the deletion mutant (Figure 1A, B). Using the external primer pair Out-PlsC-F/Out-PlsC-R, the parental strain yielded a band of approximately 901 bp. Because of the deletion, the mutant strain produced a shorter band of approximately 294 bp. The complemented strain produced two bands, corresponding to the truncated band from the original locus (294 bp) and the full-length band from the ectopically integrated Tn7 complementation (901 bp) (Figure 1A, C). These PCR results confirmed the successful construction of the *plsC* deletion and complemented strains.

### Deletion of* plsC* impairs *Brucella* growth under nutrient-restricted conditions

**Figure 2:**
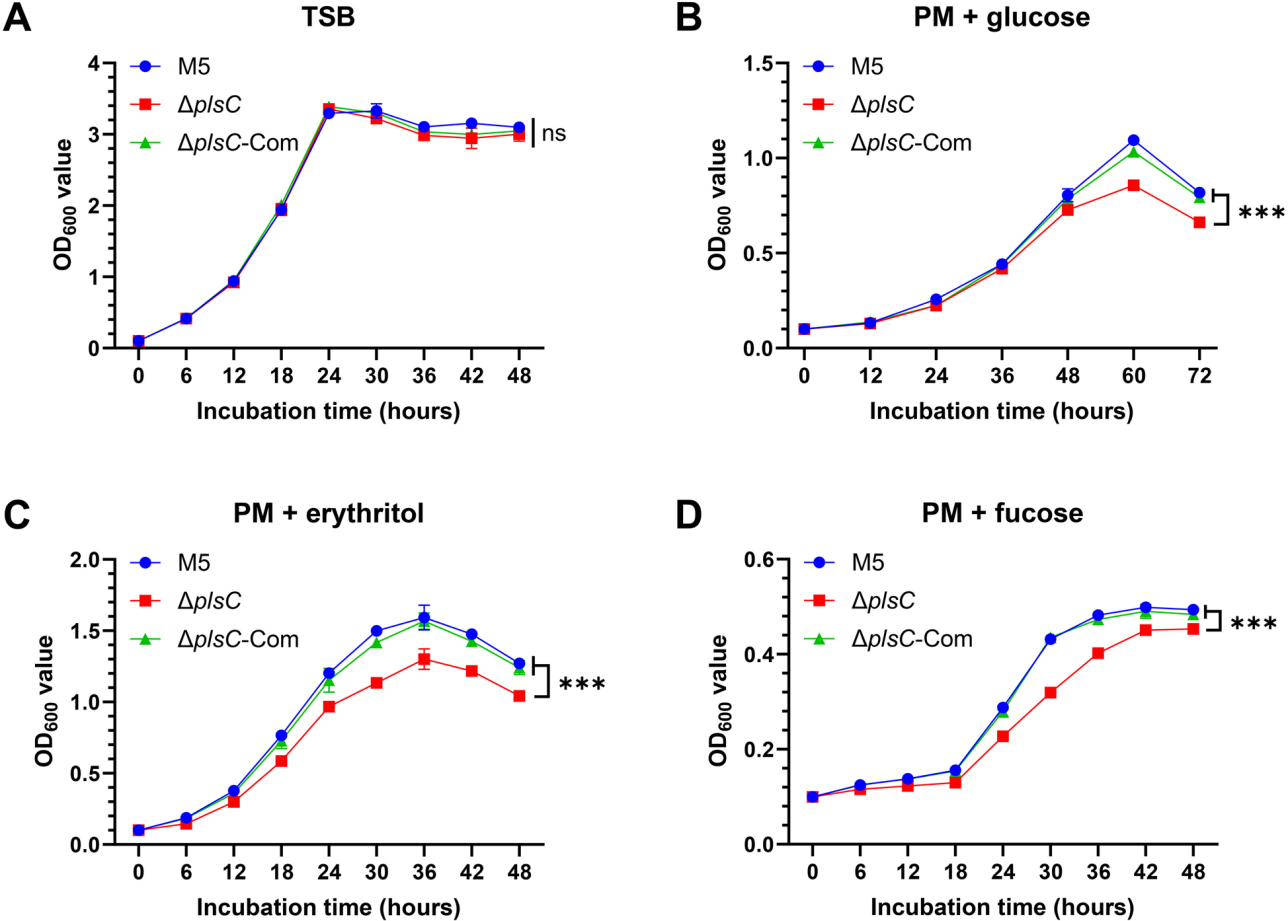
**Growth curves of *Brucella* and its derivative strains.** **A** in tryptic soy broth (TSB). **B** in chemically defined Plommet’s medium (PM) supplemented with 1 g/L D-glucose. **C** in PM with 2 g/L meso-erythritol. **D** in PM with 2 g/L L-fucose. Statistical significance was determined using two-way ANOVA (ns, not significant; ***p < 0.001).

To assess the impact of *plsC* deletion on the in vitro growth of *B. melitensis* M5, we compared the growth of the *plsC* mutant, the parental strain, and the complemented strains in nutrient-rich TSB and a chemically defined PM medium supplemented with different carbon sources. In TSB, the growth rate of the *plsC* mutant was comparable to those of the parental and complemented strains, with no significant difference (Figure 2A). However, in PM + glucose medium, the growth of the mutant was significantly reduced (Figure 2B). To determine whether this growth defect was specific to a particular carbon source, bacteria were cultured in PM medium with either erythritol or fucose as the sole carbon source. Under both conditions, the growth of the *plsC* mutant was significantly weaker than those of the parental and complemented strains (Figure 2C, D), indicating that the growth impairment caused by *plsC* deletion is not carbon source-specific. These results indicate that *plsC* deletion significantly weakens the growth capacity of *Brucella* in nutrient-poor environments.

### Deletion of *plsC* compromises *Brucella* resistance to oxidative stress and cationic antimicrobial peptides

**Figure 3:**
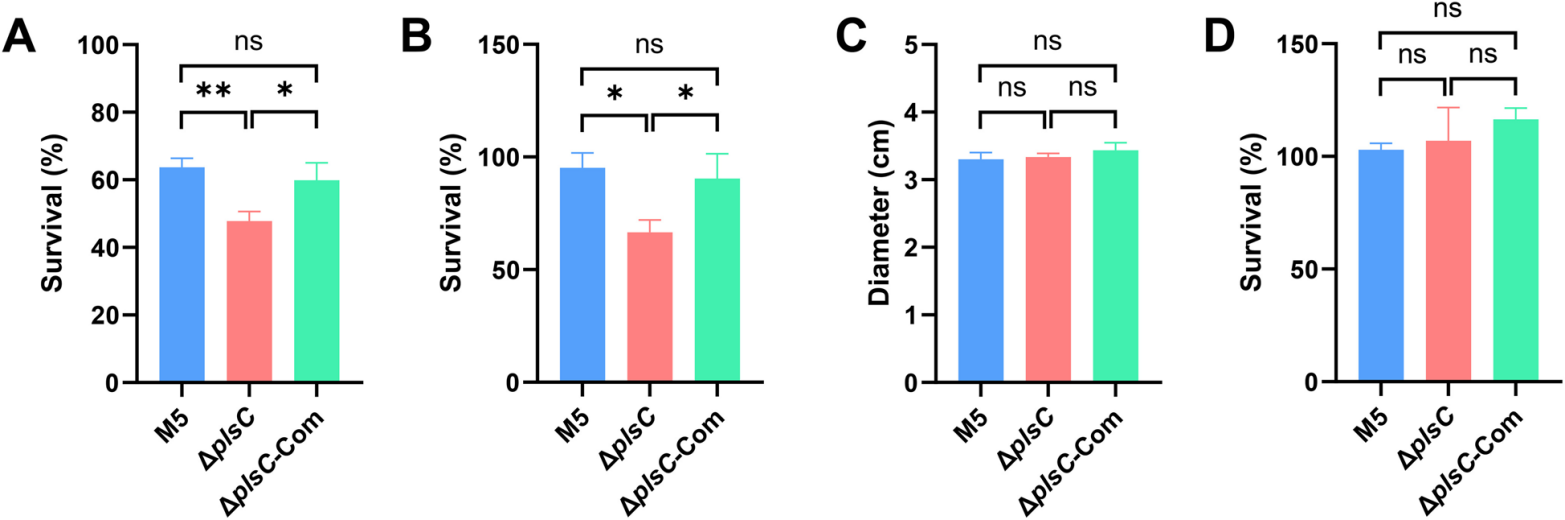
**Sensitivity of *Brucella* strains to stress factors.** **A** hydrogen peroxide. **B** polymyxin B. **C** sodium dodecyl sulfate. **D** acidic peptone water. Statistical significance was determined using one-way ANOVA (ns, not significant; *p < 0.05; **p < 0.01).

As PlsC is involved in PA synthesis, its absence may alter bacterial membrane phospholipid composition, thereby affecting membrane structural stability. To systematically evaluate the impact of *plsC* deletion on membrane integrity and stress tolerance in *Brucella*, we treated the parental, mutant, and complemented strains with H_2_O_2_ (simulating oxidative stress), polymyxin B (simulating cationic antimicrobial peptide killing), and sodium dodecyl sulfate (SDS, for assessing membrane stability), and also cultured them in acidic peptone water (pH 4.5, to simulate an acidic environment). Their survival rates under these conditions were compared. The results showed that, compared with the parental and complemented strains, the *plsC* mutant exhibited significantly reduced tolerance to both H_2_O_2_ and polymyxin B (Figure 3A, B). By contrast, no significant differences were observed among the strains when treated with SDS or under acidic conditions (pH 4.5) (Figure 3C, D). Notably, the complemented strain showed significant restoration of tolerance to both H_2_O_2_ and polymyxin B (Figure 3A, B). These findings indicate that *plsC* deletion significantly impairs the ability of *Brucella* to resist oxidative stress and cationic antimicrobial peptide killing, suggesting that PlsC plays an important role in maintaining membrane stability and environmental adaptation.

### Deletion of *plsC* affects lipid synthesis and the stability of membrane and nucleoid structures

**Figure 4:**
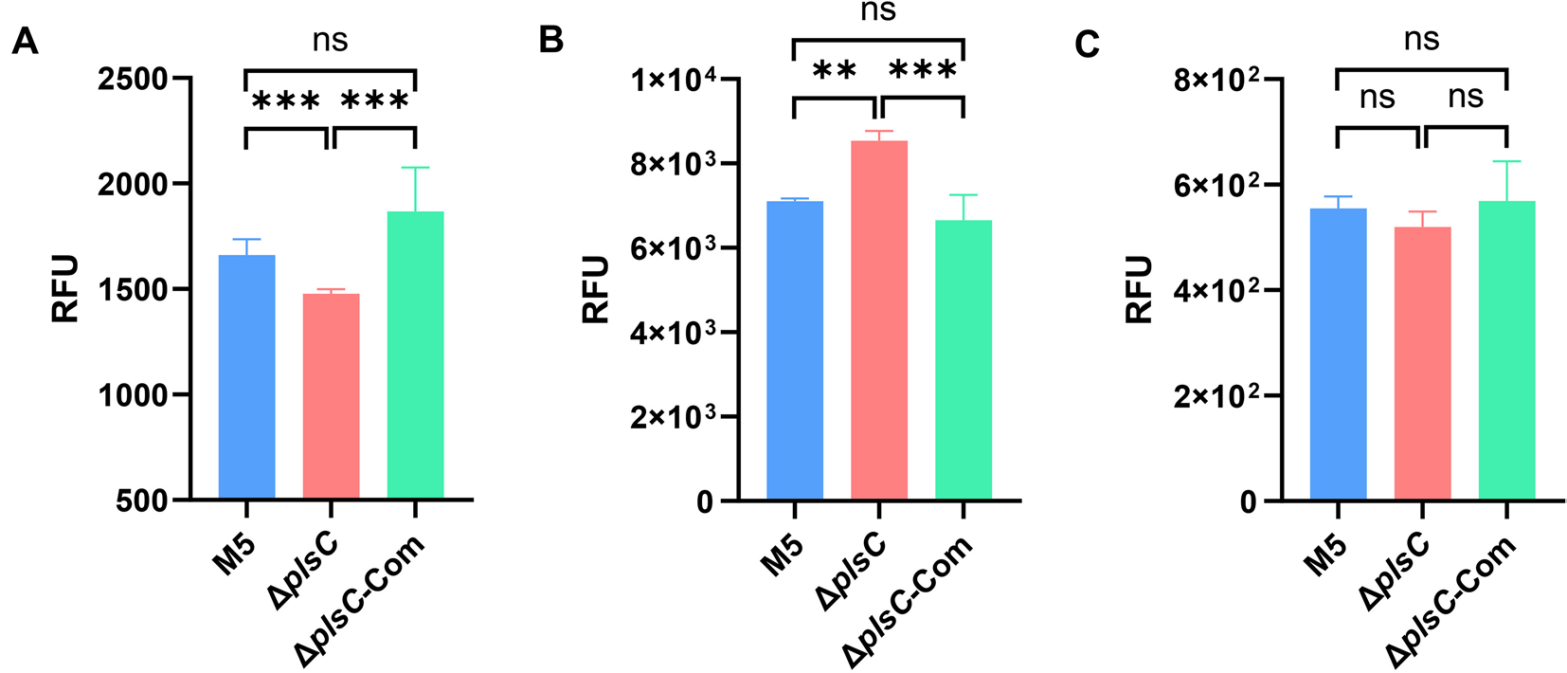
**Fluorescent dye uptake by *Brucella* and its derivative strains.** **A** Nile red. **B** 1-N-phenylnaphthylamine. **C** propidium iodide. Statistical significance was determined using one-way ANOVA (ns, not significant; ***p < 0.001;).

To investigate the effect of the *plsC* gene on lipid synthesis in *Brucella*, we assessed lipid accumulation and membrane permeability using various fluorescent dyes. NR staining revealed that the fluorescence intensity of the *plsC* mutant was significantly lower than that of the parental and complemented strains, indicating that the deletion of *plsC* markedly reduced bacterial lipid synthesis (Figure 4A). Further assessment of membrane integrity using NPN and PI staining showed that the NPN fluorescence intensity of the mutant was significantly higher than that of the parental strain, suggesting increased outer membrane permeability (Figure 4B). By contrast, PI staining showed no significant differences among the strains, indicating that inner membrane integrity remained intact (Figure 4C). Together, these results demonstrate that *plsC* deletion specifically disrupts outer membrane integrity while having minimal impact on the inner membrane.

**Figure 5:**
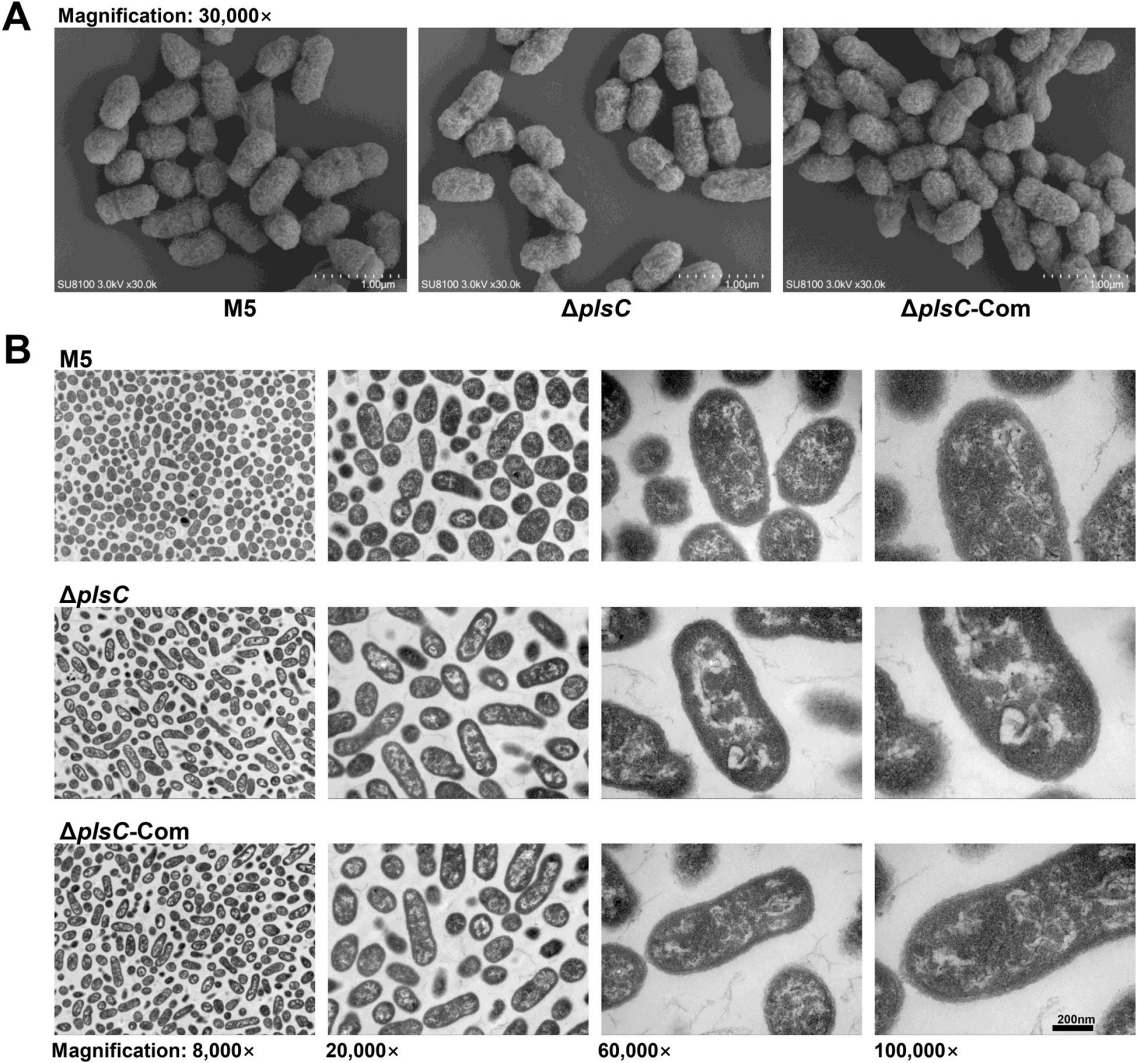
**Morphological analysis of *Brucella* and its derivative strains by electron microscopy.** **A** surface morphology visualized by scanning electron microscopy. **B** subcellular structures observed by transmission electron microscopy.

To directly observe the effects of *plsC* deletion on cellular ultrastructure, we analyzed bacterial morphology using SEM and TEM. SEM results showed that the surface of the *plsC* mutant appeared uneven and rough, whereas the complemented strain reverted to a smooth surface similar to that of parental strain (Figure 5A). TEM further revealed that although the cell membrane structure of the mutant showed no obvious alterations, numerous cytoplasmic voids were present. At higher magnification, the nucleoid region appeared condensed. These abnormalities were restored upon complementation with *plsC* (Figure 5B). These results indicate that PlsC deficiency weakens lipid synthesis capacity in *Brucella*, increases outer membrane permeability, and induces cytoplasmic voids and nucleoid structural abnormalities, thereby compromising the overall structural stability of the bacterium.

### Deletion of *plsC* attenuates *Brucella* replication within macrophages

**Figure 6:**
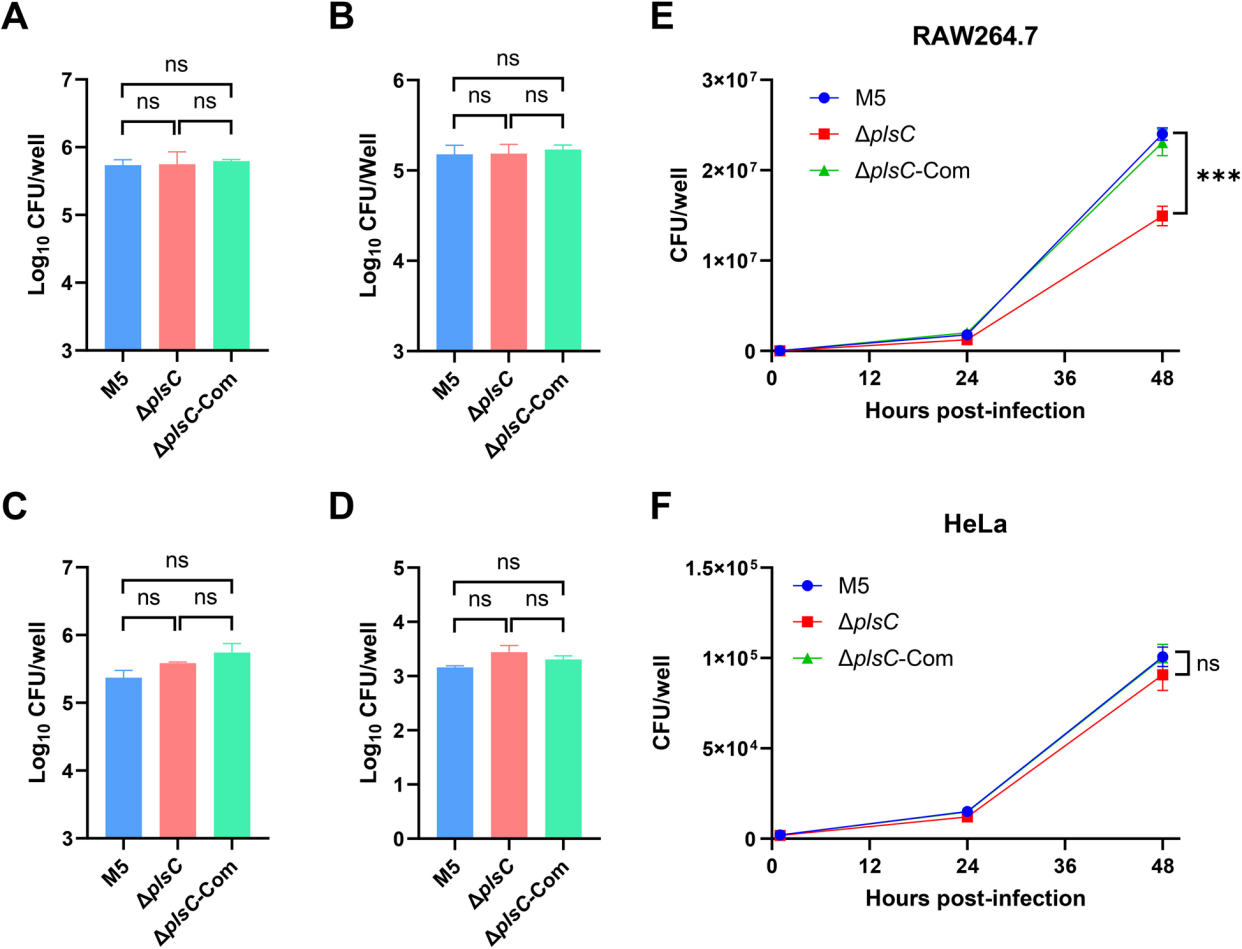
**Adhesion, invasion, and intracellular survival of *Brucella* and its derivative strains.** **A** adhesion to RAW264.7 cells. **B** invasion into RAW264.7 cells. **C** adhesion to HeLa cells. **D** invasion into HeLa cells. **E** intracellular survival in RAW264.7 cells. **F** intracellular survival in HeLa cells. Statistical significance was determined using one-way or two-way ANOVA (***p < 0.001; ns, not significant).

To evaluate the effect of PlsC deficiency on *Brucella* infectivity, we used the murine macrophage cell line RAW264.7 and the human epithelial cell line HeLa to compare the adhesion/invasion and intracellular survival of the parental, mutant, and complemented strains. Adhesion and invasion assays showed that the ability of the Δ*plsC* mutant to adhere to and invade both cell lines was not significantly affected (Figure 6A–D). Intracellular survival assays indicated that in RAW264.7 macrophages, bacterial survival levels were comparable among all strains at 24 h post-infection. However, by 48 h, the survival capacity of the Δ*plsC* mutant was significantly lower than those of the parental and complemented strains (Figure 6E). By contrast, *plsC* deletion did not significantly affect *Brucella* survival within HeLa cells (Figure 6F). These results demonstrate that PlsC deficiency specifically weakens the intracellular survival and replication capacity of *Brucella* within macrophages, without affecting its survival in nonprofessional phagocytic cells.

### Deletion of *plsC* reduces *Brucella* virulence in mice

**Figure 7:**
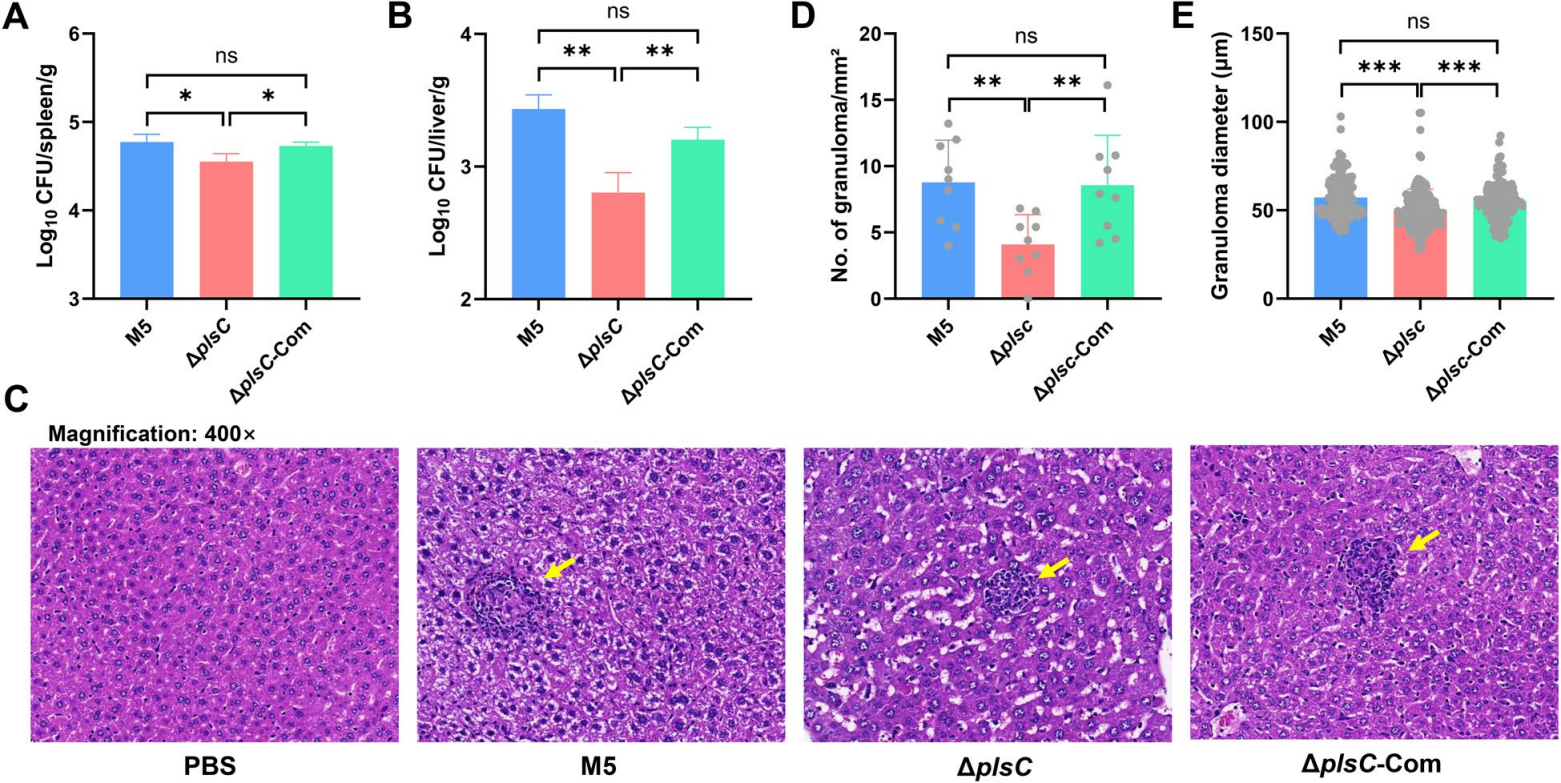
**Pathogenicity of *Brucella* strains in mice.** **A** bacterial loads in the spleen 2 weeks post-infection (wpi) with 1 × 10^6^ CFU. **B** bacterial loads in the liver at 2 wpi. **C** representative hematoxylin and eosin-stained liver sections (400 × magnification; yellow arrows indicate granulomas). **D** granuloma density per mm^2^ area. **E** granuloma diameter. Statistical significance was determined by one-way ANOVA (*p < 0.05; **p < 0.01; ***p < 0.001; ns, not significant).

To investigate the role of PlsC in *Brucella* virulence, we assessed the virulence of the parental, Δ*plsC* mutant, and complemented strains using a mouse infection model. A total of 2 weeks post-infection, the bacterial loads of the mutant in the spleen and liver were significantly lower than those of the parental and complemented strains (Figure 7A, B), indicating that PlsC deficiency significantly impairs the colonization capacity of *Brucella* in these major target organs. Liver granuloma formation is a key pathological hallmark of *Brucella* pathogenicity and of chronic infection establishment. H&E staining of liver tissue sections showed that 2 weeks post-infection, all three strains induced granuloma formation. However, the granulomas induced by the mutant were noticeably smaller than those in the control groups (Figure 7C). Further quantitative analysis revealed that both the number of granulomas per unit area (Figure 7D) and their average diameter (Figure 7E) in the mutant-infected group were significantly lower than those in the parental and complemented groups. These results indicate that *plsC* gene deletion significantly reduces the colonization capacity and granuloma-forming ability of *Brucella* in mice, thereby attenuating its virulence.

## Discussion

The study aimed to investigate the role of PlsC, a key enzyme in PA synthesis, in the pathogenicity of *B. melitensis*. Our findings indicate that the deletion of *plsC* not only disrupts lipid synthesis and membrane homeostasis in *Brucella* but also significantly impairs its carbon source utilization, intracellular survival, and virulence in mice. This work suggests the critical role of PA synthesis in *Brucella* virulence from the perspective of lipid metabolism.

To validate the function of PlsC, we successfully constructed a *plsC* gene deletion mutant and its complemented strain. PlsC catalyzes the final step in PA synthesis, and PA serves as the precursor for all major membrane phospholipids [9]. Loss of its function directly impacted lipid synthesis, as confirmed by the reduced fluorescence intensity in NR staining (Figure 4A). Growth curve analysis revealed that while the Δ*plsC* mutant showed no growth defect in nutrient-rich TSB medium, its growth was significantly attenuated in chemically defined media supplemented with glucose, erythritol, or fucose as the sole carbon source. This phenotype suggests that the growth impairment is not due to blockade of a specific carbon metabolic pathway but likely stems from a common underlying issue. Given that the cell membrane houses carbon source transporters and the energy-producing respiratory chain [15], we hypothesize that the altered membrane lipid composition caused by PlsC deletion may compromise the functional efficiency of these membrane proteins. This would lead to a general limitation in carbon uptake and energy generation, manifesting as reduced fitness under nutrient-restricted conditions. Notably, erythritol is a preferred carbon source for *Brucella* and plays a crucial role in placental tropism and the induction of abortion in pregnant animals. Its efficient utilization is closely linked to *Brucella* virulence [16–18]. Furthermore, studies have reported that glucose utilization is essential for *Brucella* to establish chronic infection in mice and to proliferate within the extracellular space of the murine placenta [19, 20]. Thus, defects in carbon source utilization provide a plausible link to the observed attenuation of in vivo virulence.

Defects in membrane lipid synthesis inevitably affect the physicochemical properties of the bacterial envelope [21]. Our study confirmed this: the mutant exhibited increased outer membrane permeability (enhanced NPN fluorescence) and showed specific hypersensitivity to stimuli mimicking host defenses—namely, significantly reduced tolerance to oxidative stress (H_2_O_2_) and the cationic antimicrobial peptide polymyxin B, while resistance to SDS and acidic environments remained unchanged. This selective sensitivity pattern is highly informative. Polymyxin B targets negatively charged LPS and membrane phospholipids [22, 23]; its enhanced bactericidal effect directly corroborates alterations in membrane surface properties or integrity. The increased sensitivity to H_2_O_2_ might be attributed either to easier penetration of harmful agents owing to membrane damage or to impairment of membrane-associated antioxidant systems (e.g., catalase activity or localization). These in vitro stress defects accurately predict the survival challenges the mutant would face within host cells.

Ultrastructural observations provided morphological evidence for the aforementioned functional defects. SEM revealed a rough surface on the *plsC* mutant, an intuitive indication of outer membrane instability. TEM further showed cytoplasmic voids and nucleoid condensation in the mutant, suggesting that defects in PA synthesis affect not only the membrane but also chromosomal structure. Such nucleoid abnormalities may result from stress responses triggered by membrane damage, energy metabolism disturbances, or ion homeostasis imbalance [24], explaining the overall physiological deterioration of the bacteria from another perspective. These structural deficiencies collectively form the cellular basis for the attenuated virulence observed in subsequent infection models.

Ultimately, all in vitro defects converged into significantly attenuated virulence in infection models. Although the mutant’s ability to adhere to and invade host cells remained intact, its survival and replication within professional phagocytes (RAW264.7 macrophages) were significantly reduced at a later infection time point (48 h). This is highly consistent with the in vitro phenotypes of hypersensitivity to oxidative stress and antimicrobial peptides, as the phagolysosomal compartment of macrophages is an extreme environment rich in reactive oxygen species and antimicrobial peptides [25, 26]. The Δ*plsC* mutant, with its compromised membrane defenses, struggled to survive persistently in this niche. By contrast, its survival was unaffected in nonprofessional phagocytes (HeLa cells), where the intracellular environment is relatively mild, further supporting the notion that its defect lies specifically in resisting host killing mechanisms. This intracellular survival defect directly led to reduced colonization capacity in vivo: bacterial loads in the spleen and liver of mice were significantly lower, and the number and size of liver granulomas—a hallmark of chronic infection—were markedly reduced. Granuloma formation relies on sustained bacterial stimulation and the recruitment and activation of immune cells [27], insufficient bacterial burden naturally attenuates this pathological process.

While these findings collectively highlight the importance of PlsC, several aspects of this study warrant consideration when interpreting the results. First, the *plsC* deletion mutant was constructed in the attenuated vaccine strain M5, not in a virulent wild‑type strain; therefore, the full extent of PlsC’s contribution to virulence in highly pathogenic *Brucella* isolates remains to be confirmed. Second, although our data indicate altered membrane permeability and lipid synthesis, we did not perform detailed lipidomic analysis to quantify specific phospholipid changes. Third, the in vivo experiments were conducted only at 2 weeks post-infection; longer‑term persistence studies would provide additional insight into the role of *plsC* in chronic infection. Despite these limitations, the consistent phenotypic defects observed across multiple assays—ranging from stress sensitivity to reduced intracellular survival and attenuated organ colonization—firmly establish PlsC as an important factor for *Brucella* membrane homeostasis and pathogenesis.

## Conclusions

This study demonstrates that PlsC deficiency disrupts lipid synthesis and compromises membrane homeostasis in *Brucella*, resulting in increased outer membrane permeability and reduced resistance to oxidative stress and antimicrobial peptides. Consequently, this defect impairs intracellular survival within macrophages, leading to diminished splenic colonization and attenuated granuloma formation in mice. Our findings not only establish the essential role of PlsC in *Brucella* physiology and pathogenicity, but also reveal, from a membrane integrity perspective, how core metabolic pathways regulate host adaptation and virulence. This work may provide a theoretical foundation for developing new antibacterial strategies directed at bacterial membrane synthesis.

## Data Availability

No datasets were generated or analyzed during the current study.
